# Factors associated with Early Initiation of Breastfeeding in Western Nepal

**DOI:** 10.3390/ijerph120809562

**Published:** 2015-08-14

**Authors:** Vishnu Khanal, Jane A. Scott, Andy H. Lee, Rajendra Karkee, Colin W. Binns

**Affiliations:** 1 Nepal Development Society, Bharatpur, 44209 Nepal; E-Mail: khanal.vishnu@gmail.com; 2 School of Public Health, Curtin University, Perth 6102, Australia; E-Mails: Andy.Lee@curtin.edu.au (A.H.L.); C.Binns@curtin.edu.au (C.W.B.); 3 School of Public Health and Community Medicine, BP Koirala Institute of Health Sciences, Dharan 56700, Nepal; E-Mail: rkarkee@gmail.com

**Keywords:** breastfeeding, cohort study, early initiation, Nepal, initiation of breastfeeding

## Abstract

The initiation of breastfeeding within one hour of birth has numerous nutritional and immunological benefits and has been found to reduce neonatal mortality. This community-based prospective cohort study aimed to report the rate of, and factors associated with, early initiation of breastfeeding in Western Nepal. The rate of early initiation of breastfeeding was reported, and associations between early initiation and independent variables were tested by Chi-square test, followed by multiple logistic regression. Of the 735 mother-infant pairs, a total of 310 (42.2%) reported early initiation. Mothers who were assisted by traditional attendants during childbirth, delivered by caesarean section, from ethnically disadvantaged families and had delivered low birth weight infants, were less likely to initiate breastfeeding early whereas the mothers who were from the poorest families and did not introduce prelacteal feeds to their infants were more likely to initiate breastfeeding within the first hour. Skills-training to support breastfeeding as part of the training of skilled birth attendants and other health workers is likely to promote recommended infant feeding practices.

## 1. Introduction

Global commitment to Millennium Development Goals has brought significant progress in child survival with an average 3.4% reduction globally in child mortality annually (1–59 months) since 1990 [1]. The progress in the reduction of neonatal mortality, however, was much slower globally (2.0% annual rate since 1990) [1]. Recent estimates show that a total of 71% of neonatal mortality could be prevented using existing antenatal, intra-partum and postnatal interventions [1]. Immediate care of the newborn which includes the nutrition of neonates (early initiation within one hour of birth and exclusive breastfeeding), is a major area of intervention for newborn survival during the perinatal period [1]. A recent meta-analysis [2] reported that the initiation of breastfeeding within 24 hours of birth was significantly associated with reduction in “all-cause neonatal mortality”, “low birth weight related neonatal mortality” and “infection related neonatal mortality” among all live births. A number of potential mechanisms for the observed reduction in mortality have been suggested [2,3] and include the early stimulation of the immune system through exposure to the high levels of immunoglobulins and lymphocytes found in colostrum, along with the displacement of prelacteal feeds which may be vehicles for infectious pathogens and also disrupt normal gut maturation, resulting in increased permeability to infectious pathogens. Furthermore, early initiation of breastfeeding is recommended as one of several steps that should be taken to prevent hypothermia in the newborn [4].

Besides reducing neonatal mortality, early initiation of breastfeeding has benefits for the mother as early suckling stimulus is linked with secretion of oxytocin which reduces the risk of postpartum haemorrhage in the mother [5]. Despite these recognised benefits of early initiation of breastfeeding, the rates in South Asia are well below universality, ranging for example from 45% [6] to 72.7% in Nepal [7], 36.4% in India [8], and 83.3% in Sri Lanka [9]. 

In Nepal, there has been significant progress in the reduction of the child mortality rate (162 per 1000 live births in 1990 to 54 in 2011) and infant mortality rate (108 per 1000 live births in 1990 to 46 in 2011) [10]. While the neonatal mortality rate decreased significantly between 2001and 2006 from 43 to 33 per 1000 live births, it remained unchanged between 2006 and 2011 [10], and the current burden of neonatal mortality is well above Nepal’s target of reducing the neonatal mortality rate to 15 per 1000 live births by 2017 [11]. While the universal adoption of early initiation of breastfeeding is likely to have a positive impact on newborn survival [12], the rates of early initiation of breastfeeding were 35% and 45% in 2006 and 2011, respectively [6]. 

Nepal is diverse in its population composition and culture, with more than 100 caste groups [13,14]. The people in the upper Himalayan region resemble the Tibetan culture whereas people in the Southern plain are culturally similar to those from India. With this heterogeneity in population and culture, infant feeding practices may vary significantly in the Western plain compared to those reported in earlier studies in central Nepal [15] or based on the 2011 Nepal Demographic and Health Survey [13] which did not include a sample from this study setting. Furthermore, appropriate infant feeding is the one of the major priorities of child health programs in Nepal [16,17]; hence, continuous monitoring of infant feeding practices is necessary to inform these programs. To date, few studies have reported factors associated with the early initiation of breastfeeding in Nepal [7,13]. The objective of this study, therefore, was to report the incidence of, and factors associated with, early initiation of breastfeeding in Western Nepal. 

## 2. Experimental Section 

A community-based cohort study was conducted in the Rupandehi district of Nepal during January–October 2014. The Rupandehi district is located in the Western plain area of Nepal bordering India to the South. This district has 69 village development committees in rural areas and two municipalities in urban areas. The village development committee and municipalities are the lowest administrative units with locally elected representatives in Nepal. The district has hospitals in the urban areas that serve as referral hospitals, and rural health facilities (primary health care centres, health posts or sub-health posts) in rural areas. Due to its plain terrain, available local area health services are relatively accessible. Nevertheless, the district had a relatively high maternal mortality rate of 274 per 100,000 live births compared to the national average of 229 per 100,000 live births for 2008/2009 [18]. 

**Figure 1:**
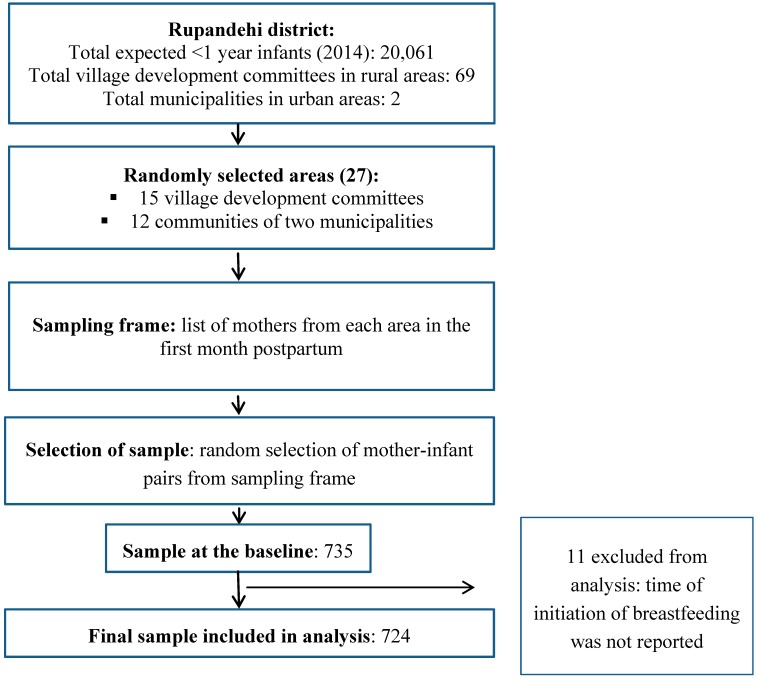
Study flow chart.

The process for recruiting the study sample is illustrated in the study flow chart (Figure 1). Briefly, the total expected district population of infants (<1 year) was 20,061 in 2014 (Personal communication District Public Health Office, Rupandehi). Mother-infant pairs were recruited from a total of 15 rural and 12 urban randomly selected communities. Lists of eligible mothers were prepared in the selected areas with the help of local female community volunteers, and health facilities. The required numbers of mothers were selected randomly from the lists. The number of mother-infant pairs recruited from each community was proportionate to population size based on the monthly target of expected numbers of infants aged <30 days. This number was calculated from the annual targets of infants based on the records of the District Public Health Office, Rupandehi. Mother-infant pairs were included if: age of the child was <30 days, mothers were residents of the community, and the child was alive at the time of recruitment. 

Data were collected by trained, experienced female enumerators who interviewed participants in their home using standardised questionnaires. We adapted the Nepali version of the questionnaire that was used in the Kaski district of Nepal [15] and which was based on questionnaires previously used in similarly designed studies of Australian women [19,20]. These questionnaires have been translated subsequently into a variety of languages for use in studies of women in Vietnam [21], China, [22,23], the Maldives [24], as well as Nepal [15]. Although the questionnaire used in the Kaski district [15] was in Nepali language which is the language spoken in our study setting, the questionnaires were pre-tested in interviews with 30 women. Some words were replaced with equivalent local terms; however, no significant changes were necessary. Baseline information and infant feeding practices were collected in the first interview.

The outcome variable for this study was “early initiation of breastfeeding” which was defined as “initiation of breastfeeding within the first hour of childbirth” based on the definition provided by the World Health Organization [25]. The variable related to the timing of breastfeeding initiation was recoded as a binary variable: “early initiation” when the newborn infant was breastfed within the first hour of birth; and “delayed initiation” when breastfed after one hour. 

Independent variables in this study were those suggested by the literature to be associated with the early initiation of breastfeeding. We categorised “assistance during delivery” as: “skilled attendance” (doctor, health assistant, nurses, auxiliary nurse midwife, auxiliary health workers, maternal and child health workers) and “unskilled/traditional attendance” (village health workers, female community health volunteer, traditional birth attendants, mother-in-law, other family members, relatives, friends, neighbours). Prelacteal feeds were defined as any fluids or foods that were provided before the introduction of first breastfeeding, and the response was recorded as “yes” or “no”. Birth weight was categorised as: “low birth weight” (<2500 g), and “average or greater” (≥2500 g or more). We categorised “maternal age” (years) as: 15–19, 20–29, and 30–45; “maternal education” as: “no education”, “primary/lower secondary” (up to grade 8), “secondary” (grade 9–10) and “higher” (grade 11–12 and university degree); “maternal occupation” as: “employed” (salaried jobs), “semi-employed” (labour and daily wage jobs), and “unemployed” (household work and subsistence agriculture work); “antenatal care visits” as: “no antenatal visit”, “1–3 visits”, and “4 or more visits”; and mode of delivery as: “vaginal”, and “caesarean” Nepal has a unique hierarchical caste-based system which is used by the government of Nepal to classify “ethnicity” [26,27]. We used this system to re-categorise the ethnic groups based on their caste similarities into: “advantaged caste groups” (Brahmin, Chhetri, Newar, Gurung, Jogi, Thakuri), “middle caste groups” (Janjati, non-Janjati and Muslim), and “Dalit caste” (Dalit). “Wealth quintiles” were derived from the scores of a principal component analysis of household assets (water source, toilet facility, types of cooking fuel, separate kitchen, floor material, electricity, radio, television, mobile phones and cupboard). The score was further divided into five quintiles ranging from the lowest (first quintile) to the highest (fifth quintile) [6,28,29]. 

### 2.1. Statistical Analyses 

The rate of early initiation was reported using frequency distribution. The associations between early initiation and independent variables were first tested using the Chi-square test. Multiple logistic regression was used to investigate the factors independently associated with early initiation of breastfeeding. All variables were entered in the initial model and the backward stepwise regression method was used to ascertain significant factors. Analysis was performed using Statistical Package for Social Science Version 20 (IBM Corp., Armonk, NY, USA). 

### 2.2. Ethical Considerations

The study protocol was approved by the Nepal Health Research Council (773/2014), and the Human Research and Ethics Committee at Curtin University (HR184/2013). Informed consent was obtained before enrolment. Mothers provided consent for themselves and their infants. 

## 3. Results 

A total of 735 mother-infant pairs were recruited with a response rate of 97.6% after 18 (2.4%) mothers declined to participate in urban areas due to possible migration. This represents approximately 22% of the total childbirths occurring in the district during the recruitment period. Characteristics of the 724 participants, who could recall the time of first breastfeeding, are presented in Table 1. Briefly, the median age of mothers was 24.0 years and about one in five had higher education (22.9%). The majority of mothers had their childbirth attended by skilled birth attendants (88.1%), and had a vaginal delivery (86.0%). A total of 96 (14.0%) of newborn infants were born with low birth weight

Of the 735 mother-infant pairs, a total of 310 (42.2%) reported early initiation; followed by breastfeeding initiation after 1 h–6 h (39.5%), after 6 h–24 h (9.8%), after 24 h–3 days (5.3%), more than three days (1.8%), or could not recall the time (n = 11, 1.3%). Of the 724 included in this analysis, the majority (92.4%) of mothers provided colostrum to their newborn infants; however, nearly one third (30.2%) were provided with prelacteal feeds that included plain water, animal milk, glucose water, honey, ghee, salt water and/or fruit juice. Of the 55 mothers who reported discarding colostrum, the major reasons were: colostrum is difficult to digest (n = 23, 41.8%), not clean (n = 20, 36.3%), harmful to baby (n = 2, 3.6%), and does not look nice (n = 3, 5.45%). 

### Factors Associated with Early Initiation of Breastfeeding

The results of the stepwise multivariable logistic regression (Table 2) showed that being assisted by a traditional birth attendant was significantly associated with lower likelihood of early initiation of breastfeeding (adjusted odds ratio (aOR): 0.47, 95% confidence interval (CI): 0.22–0.99). Infants who were born by caesarean section (aOR: 0.39; 95% CI: 0.239–0.67), born to middle caste group (aOR: 0.62; 95% CI: 0.40–0.95) and Dalit caste group (aOR: 0.52; 95% CI: 0.28 to 0.94) were less likely to be breastfed within the first hour of their life. In addition, the infants who were born low birth weight (aOR: 0.36; 95% CI: 0.21–0.63) and born to mothers of older age *i.e.*, 30–45 years (aOR: 0.45, 95% CI: 0.22–0.93) were less likely to be breastfed within one hour of birth. Infants born to the poorest families (aOR: 2.43; 95% CI: 1.36–4.37) were more likely to be breastfed within one hour of childbirth. Infants who were not provided prelacteal feeds had higher likelihood of breastfeeding initiation within one hour of birth (aOR: 2.00; 95% CI: 1.35–2.98).

**Table 1:** Characteristics of participants according to time to first breastfeed in western Nepal.

Factor	Frequency *n* (%)	Time to First Breastfeed		*p* Value *
		Delayed *n* (%)	Early *n* (%)	
**Maternal age (in years) ^#^ (*n* = 723)**				0.003
15–19	64 (8.9)	30 (46.9)	34 (53.1)	
20–29	542 (75.0)	301 (55.5)	241 (44.5)	
30–45	117 (16.2)	82 (70.1)	35 (29.9)	
**Maternal education (*n* = 724)**				0.208
No education	190 (26.2)	119 (62.6)	71 (37.4)	
Primary/Lower secondary	240 (33.1)	129 (53.8)	111 (46.2)	
Secondary	128 (17.7)	68 (53.1)	60 (46.9)	
Higher	166 (22.9)	98 (59.0)	68 (41.0)	
**Maternal occupation (*n* = 724)**				0.001
Employed	30 (4.2)	17 (56.7)	13 (43.3)	
Semi-employed	140 (19.3)	99 (70.7)	41 (29.3)	
Unemployed	554 (76.5)	298 (53.8)	256 (46.2)	
**Antenatal care visits ^#^ (*n* = 721)**				0.669
No antenatal visit	17 (2.4)	11 (64.7)	6 (35.3)	
1–3 visits	155 (21.5)	92 (59.4)	63 (40.6)	
4 or more visits	549 (76.1)	310 (56.5)	239 (43.5)	
**Assistance during delivery (*n* = 724)**				0.069
Unskilled/Traditional attendants	86 (11.9)	57 (66.3)	29 (33.7)	
Skilled attendants	638 (88.1)	357 (56.0)	281 (44.0)	
**Mode of delivery (*n* = 724)**				<0.001
Vaginal	623 (86.0)	338 (54.3)	285 (45.7)	
Caesarean	101 (14.0)	76 (75.2)	25 (24.8)	
**Ethnicity (*n* = 724)**				0.481
Advantaged caste group	270 (37.3)	149 (55.2)	121 (44.8)	
Middle caste groups	366 (50.6)	210 (57.4)	156 (42.6)	
Dalit caste	88 (12.2)	55 (62.5)	33 (37.5)	
**Wealth quintile (*n* = 724)**				<0.001
1 Lowest	146 (20.2)	61 (41.8)	85 (58.2)	
2	146 (20.2)	84 (57.5)	62 (42.5)	
3	145 (20.0)	85 (58.6)	60 (41.4)	
4	146 (20.2)	98 (67.1)	48 (32.9)	
5 Highest	141 (19.5)	86 (61.0)	55 (39.0)	
		Delayed *n* (%)	Early *n* (%)	
**Sex of infant (*n* = 724)**				0.520
Male	379 (52.3)	221 (58.3)	158 (41.7)	
Female	345 (47.7)	193 (55.9)	152 (44.1)	
**Birth order ^#^ (*n* = 723)**				0.062
First	307 (42.5)	170 (55.4)	137 (44.6)	
Second or third	329 (45.5)	184 (55.9)	145 (44.1)	
Fourth or more	87 (12.0)	60 (69.0)	27 (31.0)	
**Birth weight ^#^ (*n* = 668)**				<0.001
Low birth weight (<2500 g)	93 (14.0)	72 (77.4)	21 (22.6)	
Average or greater (≥2500 g)	573 (86.0)	311 (54.3)	262 (45.7)	
**Place of residence (*n* = 724)**				0.577
Rural	373 (51.5)	217 (58.2)	156 (41.8)	
Urban	351 (48.5)	197 (56.1)	154 (43.9)	
**Prelacteal feeds (*n* = 724)**				<0.001
Not provided	505 (69.8)	252 (49.9)	253 (50.1)	
Provided	219 (30.2)	162 (74.0)	57 (26.0)	

*: *p* value: Chi-square test *p*-value; **^#^**: the total in each variable varies due to missing responses; Early: initiation of breastfeeding within 1 h of birth; Delayed: initiation of breastfeeding after one hour of birth.

**Table 2:** Factors associated with early initiation of breastfeeding in Western Nepal.

Factors	Crude Odds Ratio (95% Confidence Interval)	*p*-Value	Adjusted Odds Ratio (95% Confidence Interval)	*p*-Value
**Maternal age (in years)**		0.004		0.040
15–19	1.00		1.00	
20–29	0.71 (0.42, 1.19)		0.83 (0.46, 1.50)	
30–45	0.38 (0.20, 0.71)		0.45 (0.22, 0.93)	
**Assistance during delivery**		0.071		0.048
Unskilled/Traditional attendant	0.65 (0.40, 1.04)		0.47 (0.22, 0.99)	
Skilled attendant	1.00		1.00	
**Mode of delivery**	*p* < 0.001		*p* = 0.001	
Vaginal	1.00		1.00	
Caesarean	0.39 (0.24, 0.63)		0.39 (0.23, 0.67)	
**Ethnicity**		0.483		0.038
Advantaged caste group	1.00		1.00	
Middle caste groups	0.91 (0.67, 1.26)		0.62 (0.40, 0.95)	
Dalit caste	0.75 (0.45, 1.21)		0.52 (0.28, 0.94)	
**Wealth quintile**		0.001		0.001
1 Lowest	2.18 (1.36, 3.49)		2.43 (1.36, 4.37)	
2	1.15 (0.72, 1.85)		1.36 (0.77, 2.40)	
3	1.10 (0.69, 1.77)		1.22 (0.72, 2.06)	
4	0.77 (0.47, 1.24)		0.69 (0.41, 1.15)	
5 Highest	1.00		1.00	
**Birth weight**		<0.001		<0.001
Low birth weight (<2500 g)	0.35 (0.21, 0.58)		0.36 (0.21, 0.63)	
Average or greater	1.00		1.00	
**Prelacteal feeds**		<0.001		<0.001
Not provided	2.85 (2.01, 4.04)		2.00 (1.35, 2.98)	
Provided	1.00		1.00	

Variables excluded during backward stepwise regression: maternal education, maternal occupation, antenatal care visits, sex of infant, birth order, place of residence.

## 4. Discussion

This study showed that only about four in 10 infants were being breastfed within the first hour of birth. The finding is comparable to the recent Nepal Demographic and Health Survey 2011 (45%), but, much lower than that reported for the Kaski district (67%) in central Nepal [15] and higher than that reported by the 2006 Nepal Demographic and Health Survey (35.4%) [29]. Our finding is higher than those reported for neighboring countries Bangladesh (24%) [30], and India (36.4%) [8] but lower than that of Sri Lanka (83.3%) [9]. Such variation in the rates of early initiation within Nepal and within the South Asian countries is likely due to differences in the geography, ethnicity, culture and socioeconomic status of populations [13].

A high neonatal mortality rate of 33 per 1000 live birth is a major concern for child survival in Nepal, and neonatal mortality was identified as one of the priority areas of action in the recent Millennium Development Goals country progress report (2013) [10] and the National Safe Motherhood and Newborn Health Long-Term Plan (2006–2017) [11]. Immediate newborn care, including early nutrition interventions such as early initiation and exclusive breastfeeding are some of the proven interventions that reduce neonatal mortality [1]. Debes *et al.* [2] have shown that the initiation of breastfeeding within 24 hours of birth can reduce up to 44% of all cause neonatal mortality, and 42% of low birth weight related neonatal mortality. An earlier Nepali study also found that initiation of breastfeeding within 24 hours of birth can prevent about 19.1% of all neonatal deaths [31]. A Ghanaian study further reported an increase in the risk of neonatal mortality with delayed breastfeeding initiation [3] with the highest risks being associated with breastfeeding being initiated after three days (aOR: 3.64; 95% CI: 1.43, 9.30) compared to that initiated within an hour. Increasing the rate of early initiation of breastfeeding from the current rate of 42.2% to universal practice (near 100%), therefore, is likely to contribute significantly to reducing neonatal mortality in Nepal.

Prelacteal feeding, which in Nepal includes honey, ghee (refined butter), sugar water and animal milk, was associated with delayed initiation of breastfeeding. Just under one third of infants received prelacteal feeds and of these three quarters were not breastfed within the first hour. A similar association between prelacteal feeding and delayed initiation of breastfeeding was reported also by studies in the southern region of Nepal [32] and India [8]. As this is a cross-sectional analysis, it is not clear whether prelacteal feeding is a cause or consequence of delayed initiation. 

The Ministry of Health and Population in Nepal trains health workers to become skilled birth attendants. As part of the curriculum, skilled birth attendants are trained in ‘supporting successful breastfeeding’ that includes ‘education’, and ‘skill’ components on encouraging and supporting mothers to breastfeed as part of immediate newborn care [33]. Our findings showed that being assisted by skilled attendants during childbirth increased the likelihood of a woman initiating breastfeeding within one hour of childbirth, which is consistent with the skilled attendants’ training. Previous findings from Nepal [13] and Sri Lanka [9] have also reported that the mothers who had their childbirth in a health facility, attended by health workers, were more likely to initiate breastfeeding within the first hour of birth. 

This study found that low birth weight newborn infants were more at risk of delayed breastfeeding. This finding is similar to those from Sri Lanka [9] where low birthweight newborns had less likelihood of being breastfed within the first hour of birth. This could be due to poor suckling capacity or associated illness among the low birth weight infants [8]. A high prevalence of low birth weight infants is one of the challenges to neonatal survival in Nepal [16]. Indeed, low birth weight infants need immediate breastfeeding [34], which is helpful in reducing hypothermia [16,35] as their bodies are less able to self-regulate body temperature [36]. Low birth weight infants have greater benefits from early initiation of breastfeeding as breast milk protects from necrotising enterocolitis, milk intolerance, and early onset of sepsis among these groups [34]. Therefore, future breastfeeding promotion programs should focus on immediate breastfeeding of low birth infants. In addition, small newborns need support for feeding; therefore, nurses and health workers must receive training in the care and support of low birth weight newborns [36]. 

Caesarean delivery has been reported to be a major risk factor of lower duration of exclusive breastfeeding, delayed initiation of breastfeeding [8,37] and increased risk of prelacteal feeding [15]. Our study also found that the infants who were delivered by caesarean section were also at risk of not being breastfed within the first hour of birth. The effect of anaesthesia, caesarean procedure, maternal tiredness, reduced maternal alertness and inadequate maternal skills to initiate breastfeeding are some of the reasons for delayed breastfeeding among caesarean births [8]. 

Maternal socio-demographic factors have been reported to be important factors that determine infant feeding behaviour. In our study, two socio-demographic factors were also found to be significantly associated with time of initiation of breastfeeding. Mothers who were from the lowest wealth quintile were most likely to initiate breastfeeding in the first hour of childbirth, similar to those reported from Sri Lanka [9]. Mothers from the lowest wealth quintile might not have access to infant formula or have less capacity to buy expensive breast milk substitutes such as honey and ghee (refined butter), that could leave breastfeeding as the only choice. On the other hand, the mothers from the highest wealth quintile may be more likely to have an elected caesarean section, leading to delayed initiation of breastfeeding [37]. However, further qualitative studies are needed to explore why the mothers from the highest wealth quintile tended to delay initiation of breastfeeding. 

Surprisingly, mothers who identified themselves as marginalised Dalit caste group were more likely to delay initiation of breastfeeding. Nepal has unique caste-based ethnic groups in which the Dalit caste constitutes the lowest rank in the caste hierarchy of Nepali society. While there is no overt government discrimination restricting this caste group’s access to health care and other services, members of this caste traditionally have been socially excluded and, for example, restricted from entering temples and water sources, resulting in a subordinate social hierarchy and poor health status [27,38]. While there is limited literature on infant feeding practices related to disadvantaged ethnic groups of Nepal, our findings suggest that infants born to these families are vulnerable to delayed initiation of breastfeeding. Further study is necessary to explore the cultural reasons for why the Dalit ethnic group has a higher risk of delayed initiation independent of socio-economic status.

This study is one of the few studies that report on early initiation of breastfeeding in the plain areas of Western Nepal. The recall period for this study is short compared to previously published studies [13,15]; therefore, recall bias is minimal and the measurement of early initiation is more accurate. A limitation of this study is the small number of mothers (n = 11) who could not report the time of initiation of breastfeeding; however, this is unlikely to change our result. This study reports only on the South-western plain area of Nepal; therefore, the findings may not be generalizable to the entire country due to cultural and ethnic difference in other parts of the country. Nevertheless, the findings of this study are generally consistent with those of studies in other regions in Nepal and countries in the region. Hence, the results may be used to inform both local and national infant nutrition programs as the findings provide useful insights into those groups most at risk of delayed breastfeeding initiation, as well as potentially modifiable risk factors for this practice. 

## 5. Conclusions 

This study demonstrates that delayed initiation of breastfeeding continues to be a problem in Nepal as only four in 10 newborn infants received breast milk within the first hour of birth. Given the protective association of early breastfeeding and neonatal mortality, promoting early initiation of breastfeeding at a universal level will contribute to significant progress in newborn survival in the post Millennium Development Goal period in Nepal. The results show that receiving assistance from skilled birth attendants during childbirth had a positive impact on early infant feeding practices. The skilled attendance at childbirth is increasing in Nepal; therefore, including lactation support skills as part of the training of skilled birth attendants and other health workers, who provide support during childbirth, is likely to promote recommended infant feeding practices. Those mothers who are from disadvantaged ethnic groups, deliver by caesarean section and deliver low birth weight infants should be targeted when implementing breastfeeding promotion programs. 
